# 3D Light-Direction Sensor Based on Segmented Concentric Nanorings Combined with Deep Learning

**DOI:** 10.3390/mi15101219

**Published:** 2024-09-30

**Authors:** Pengcheng Huang, Peijin Wu, Ziyuan Guo, Zhicheng Ye

**Affiliations:** 1 Department of Electronic Engineering, Shanghai Jiao Tong University, Shanghai 200240, China; pchhuang@sjtu.edu.cn (P.H.); guo.zy@sjtu.edu.cn (Z.G.); 2 Key Laboratory for Laser Plasmas (Ministry of Education), School of Physics and Astronomy, Shanghai Jiao Tong University, Shanghai 200240, China; wupeijin@sjtu.edu.cn; 3 Fujian Science & Technology Innovation Laboratory for Optoelectronic Information of China, Fuzhou 350108, China; 4 Hangzhou Institute of Optics and Fine Mechanics, Hangzhou 311421, China

**Keywords:** 3D light-direction sensor, deep learning, nanorings, light-field reconstruction

## Abstract

High-precision, ultra-thin angular detectable imaging upon a single pixel holds significant promise for light-field detection and reconstruction, thereby catalyzing advancements in machine vision and interaction technology. Traditional light-direction angle sensors relying on optical components like gratings and lenses face inherent constraints from diffraction limits in achieving device miniaturization. Recently, angle sensors via coupled double nanowires have demonstrated prowess in attaining high-precision angle perception of incident light at sub-wavelength device scales, which may herald a novel design paradigm for ultra-compact angle sensors. However, the current approach to measuring the three-dimensional (3D) incident light direction is unstable. In this paper, we propose a sensor concept capable of discerning the 3D light-direction based on a segmented concentric nanoring structure that is sensitive to both elevation angle (θ) and azimuth angle (ϕ) at a micrometer device scale and is validated through simulations. Through deep learning (DL) analysis and prediction, our simulations reveal that for angle scanning with a step size of 1°, the device can still achieve a detection range of 0∼360° for ϕ and 45°∼90° for θ, with an average accuracy of 0.19°, and DL can further solve some data aliasing problems to expand the sensing range. Our design broadens the angle sensing dimension based on mutual resonance coupling among nanoring segments, and through waveguide implementation or sensor array arrangements, the detection range can be flexibly adjusted to accommodate diverse application scenarios.

## 1. Introduction

Information on the incident angle is crucial for light-field detection and reconstruction [1]. Relying on the angle information, wavefront detection, dynamic ranging, and image refocusing can thus be implemented [2,3,4], and these capabilities have significant implications in machine vision, virtual reality (VR), and augmented reality (AR) [5]. The key to designing angle sensors lies in converting the incident angle information into other corresponding measurable physical quantities with high angular sensibility. Generally, physical effects with angular sensitivity include light projection and masking, the Talbot effect, and the resonance coupling mechanism [6].

Specifically, light projection and masking refer to angle detection based on the dependence of the projection area on the incident angle of light rays [7,8]. A typical example is the four-quadrant detector (4-QD) [9], which determines the angle of incident light by calculating the proportion of light currents among the four different quadrants. The Talbot effect describes the self-imaging property of periodic objects such as diffraction gratings, and the shifts or the depth of self-images are sensitively changeable by variation in the angle for incident light. Through this effect, incident angles can be obtained according to the changing rules of the self-images [10]. However, angle sensors that rely on lenses or gratings are limited by diffraction theory [11]. When a sensor’s scale is close to or smaller than the wavelength of the detection light, its accuracy will sharply decrease due to the minimal recognizable half wavelength limits between two adjacent imaged spots, and this makes it difficult to achieve the design goal of ultra-thin sensors with deep miniaturization.

As for resonance coupling, it typically occurs in sub-wavelength structures or devices such as nanowires and surface plasmon, it describes the interaction of structures under excitation, and its characteristics are tightly related to the incentives. Through elaborate design and testing, the resonance can be used for angle detection, which can be deduced by measurable physical quantities, e.g., photocurrents [12,13]. And to address the challenge of miniaturization on light-direction sensors, inspired by the directional sensing ability of small animals to sound and light waves, angle sensors based on coupled nanowires have demonstrated the high-precision angle perception of incident light at sub-wavelength scales and can be manufactured with existing mature nanofabrication processes [14,15,16,17].

Perovskite nanocrystals have excellent optoelectronic properties and can produce adjustable emissions with high color saturation under specific spectra, which can convert light-field information into color output. By arranging multicolor-emitting perovskite nanocrystals geometrically, the incident light angles can be calculated by decoding the color output, which utilizes the mutual occlusion between the nanocrystals under angular irradiation [18].

However, the above-mentioned incident light angle measurement methods are limited to planar angle measurement and belong to the two-dimensional (2D) level. Comprehensive detection of three-dimensional (3D) light-direction, including the elevation angle (θ) and azimuth angle (ϕ), typically relies on a combination of sensors, which often entails a compromise between a sensor’s detection ability and physical size. Although the perovskite nanocrystal-based method is capable of sensing 3D light direction, its large-scale application is relatively costly and complex, and exploring new design mechanisms is necessary.

In order to overcome the miniaturization dilemma caused by diffraction limits and achieve comprehensive detection of 3D light-direction angles, in this work, we propose a 3D light-direction sensor unit based on the angular dependency of the resonant energy distribution within segmented concentric nanorings, capable of detecting incident light-direction upon a single device. Furthermore, a deep learning (DL) method is employed to fit and reason the calculation relationship between the angles and the proposed structural responses; thus, the trained data-driven model can be used to obtain angles. Additionally, the fabrication and measurement feasibility of the proposed sensor are shown. In terms of application, the proposed detector can be integrated with devices such as lenses and waveguides to enhance application flexibility and improve detection performance.

## 2. Materials and Methods

### 2.1. Nanoring Resonance for Angle Sensing

**Figure 1:**
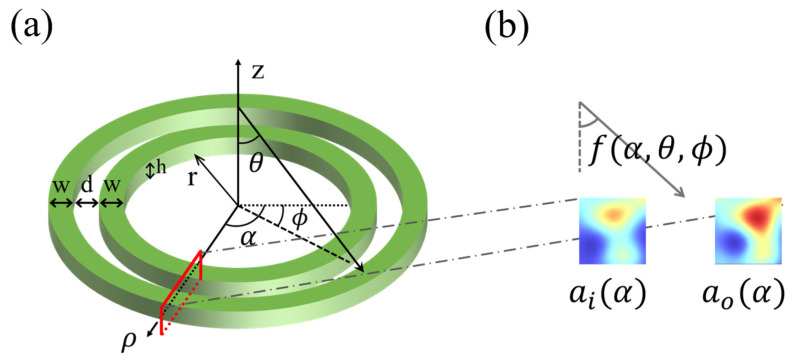
(**a**) Structural diagram of double concentric Si-nanoring resonators. (**b**) For the cross-sections determined by the coordinate angle α in cylindrical coordinates (ρ,α,z), the effective angle of incident light received is a function of (θ,ϕ,α).

Paired resonant nanowires with a high refractive index have been theoretically and experimentally demonstrated to exhibit the strong angular sensitiveness of leaky-mode resonance, deeming are suitable for angle sensors [12]. Similarly, as a commonly used coupled waveguide device, concentric nanorings can couple and resonate with 3D light-direction angular dependence under incident light irradiation [19]. For the concentric silicon nanorings shown in Figure 1a, nanorings with the same width (w) and height (h) are arranged concentrically with a spacing (d), and the radius of the inner nanoring is defined as r.

When space light is incident with the 3D direction of (θ,ϕ), optical Mie resonances are supported within the inner and outer nanoring cross-sections $a_{i}(\alpha )$ and $a_{o}(\alpha )$ determined by the coordinate angle α in cylindrical coordinates (ρ,α,z) (Figure 1b), whose energy distribution can be modeled as follows [20,21,22,23,24,25]:

(1) iddtai(α)ao(α)=H0(θ,ϕ)ai(α)ao(α)+Hi(θ,ϕ)ai(α)ao(α)             +iκf(α,θ,ϕ)S(f(α,θ,ϕ))exp⁡(−iπsin⁡f(α,θ,ϕ)d/λ)exp⁡(iπsin⁡f(α,θ,ϕ)d/λ)

where ${\left|a_{i}\left(\alpha \right)\right|}^{2}$, ${\left|a_{o}\left(\alpha \right)\right|}^{2}$ represent the stored energy in the inner and outer nanoring cross-sections determined by α, and $f\left(\alpha ,\theta ,\phi \right)=\theta \cdot \cos\left(\alpha -\phi \right)$ is the effective angle of incident light received by the cross-sections. $H_{0}\;$and $H_{i}(\theta ,\phi )$ are the usual Hamiltonian for a pair of resonators and the non-Hermitian Hamiltonian for treating the far-field couple in leakage channels, respectively. The flux S(f(α,θ,ϕ)), denoting the incident light energy pumped into the resonators, and the coupling rate$\;\kappa_{f\left(\alpha ,\theta ,\phi \right)}$ are the main contributors to the angular dependency. The two quantities are determined by the 3D light-direction angles (θ,ϕ), which control the phase $\mp \frac{i\pi cos(f(\alpha ,\theta ,\phi )))d}{\lambda }$ of the excitation wave at each cross-section. Furthermore, the total energy stored in the segments of the inner and outer nanorings determined by the range of $\alpha \in (\alpha_{1},\alpha_{2})$ can be calculated as follows:

(2) $$e_{i}=\int_{\alpha_{1}}^{\alpha_{2}}|a_{i}(\alpha ){|}^{2}d\alpha ;e_{o}=\int_{\alpha_{1}}^{\alpha_{2}}|a_{o}(\alpha ){|}^{2}d\alpha$$

**Figure 2:**
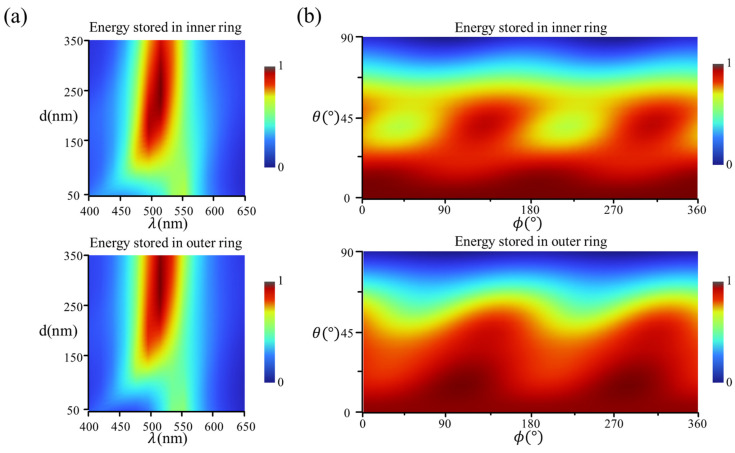
(**a**) Maps of the normalized absorption intensity of the inner and the outer nanorings with spacing (d) and wavelength (λ) under normal incident light; (**b**) numerical maps of the normalized absorption intensity of the inner and the outer nanorings for different (θ,ϕ).

The ratio of the inner and outer energy $r=e_{i}/e_{o}$ is a function of (θ,ϕ), from which the 3D light-direction angular sensitivity physical quantity is obtained. For complete concentric nanorings, the response characteristics are polarization-independent due to the strict pairwise nature of the structure, and the energy stored in the inner and outer ring differs from the spacing d and the wavelength of incident light λ, as shown in Figure 2a with the normal incident at s-polarization. In this paper, d is set as 100 nm and λ is set as 550 nm within the absorptive band of Si material to ensure moderate stored energies in both the inner and outer rings for the ratio comparison analysis and actual detection, as shown in Figure 2a. Meanwhile, the (θ,ϕ) influences the resonances within the inner and outer nanorings and further determines the energy stored, as shown at the top and bottom of Figure 2b, respectively.

### 2.2. Three-Dimensional Light-Direction Sensor

**Figure 3:**
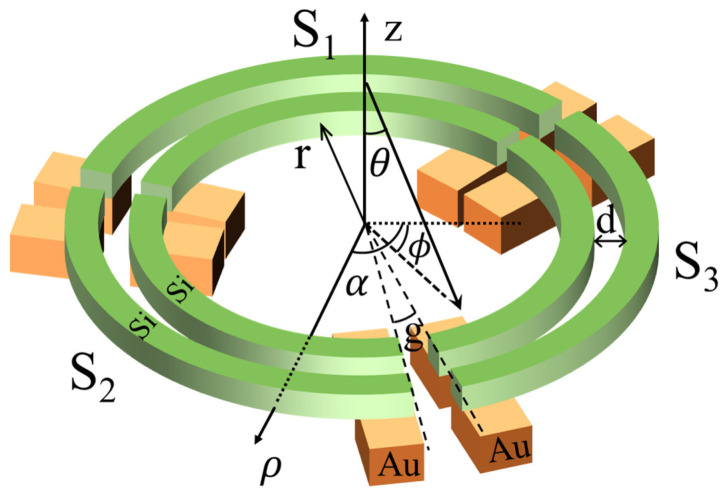
Structural diagram of the 3-segment double-concentric nanoring sensor. Two nanorings are divided into inner and outer rings and further segmented into three pairs: *S*_1_, *S*_2_, and *S*_3_. The inner radius of the nanoring (r) is 1000 nm, the width (w), height (h) of nanorings, and the spacing (d) are 100 nm, and the gap between adjacent nanoring pairs that corresponds to a central angle (g) is 5°.

To achieve 3D light-direction sensing with a single pair, the response of its structure must exhibit continuous, sensitive, uniform, and monotonous dependence on both the θ and the ϕ simultaneously within the detection range. Due to circular symmetry, the energy stored in the complete nanoring is independent of ϕ. Thus, it is necessary to segment the nanorings to introduce azimuth angle sensitivity, and for full ϕ sensing, at least three segments are needed, as proposed in Figure 3. Assuming that the nanorings are segmented into three pairs—*S*_1_ (α∈(0,120°)), *S*_2_ (α∈(120°,240°)), and *S*_3_ (α∈(240°,360°))—the energy stored in the inner and outer segments within the same pair can be calculated as follows:

(3) $$e_{i_{j}/o_{j}}=\int_{S_{j}}|a_{i_{j}/o_{j}}(\alpha ){|}^{2}d\alpha ,\;j=1,2,3$$

where the energy stored in pairs *S*_1_, *S*_2_, *S*_3_ is $S_{j}=e_{i_{j}}+e_{o_{j}}$. According to the equations derived above, physical quantities of energy distribution exhibiting strong angle correlations can be induced, which include the ratio of energy between the inner and the outer segment within the same pair ($e_{i_{j}}/e_{o_{j}}$) and the ratio of energy stored by different pairs $\left(S_{j}/S_{\left(j+1\right)mod\;3}\right)$. Both quantity ratios demonstrate a strong functional dependence on the angles (θ,ϕ), enabling accurate 3D light-direction angle calculation, which will be elaborated in detail in the discussion section.

In working, the energy in the resonator’s segments can be further quantified as photocurrent responses. Thus, the incident angle can then be calculated by the photocurrents exported from the metal (Au/Al) electrodes of the nanoring segments [26]. Additionally, considering the practical working condition and stability of the devices, the structure was fabricated on a silicon dioxide (SiO_2_) substrate and most of the architecture, excluding the metal electrodes, was protected by SiO_2_ to afford resistance to temperature and humidity.

In summary, the principle of high sensitivity for the segmented concentric nanorings is similar to the angular dependence expounded in the parallel straight nanowires [12]. The non-Hermitian coupling between the inner and outer segments of the same nanoring pair results in a strong angular dependence on elevation. Therefore, θ can be calculated by the ratio of photocurrents between these two segments (RPS). Meanwhile, the differences in responsivities among the nanoring pairs enable azimuthal determination, as the resonance in each pair especially depends on the azimuth. Consequently, ϕ can be calculated by the ratio of total photocurrents between adjacent nanoring pairs (RPPs). Through reasonable adjustments, including the number of segments and structural parameters, higher-precision detection of the 3D light-direction angles can be obtained. Moreover, the proposed structure behaves with high symmetry to ensure translational responses to the polarization of the incident light, indicating computational universality.

**Figure 4:**
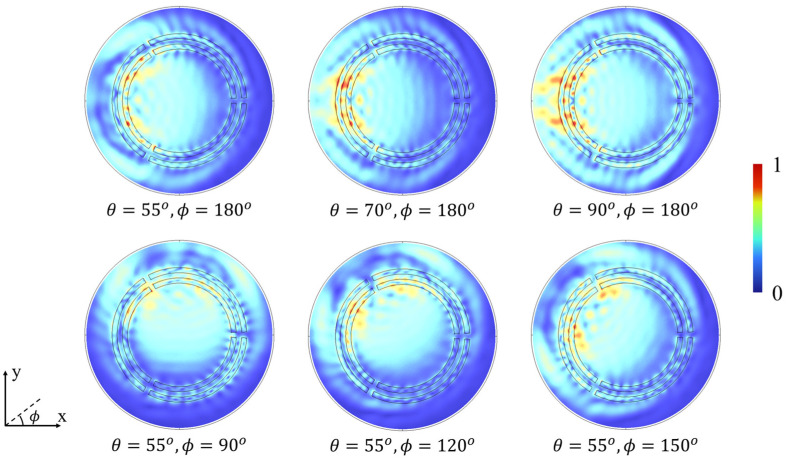
The normalized electric field distribution map for different 3D light-direction angles.

For the numerical simulations of light energy absorption at different (θ,ϕ) in the proposed segmented concentric nanorings, the 3D finite element method (FEM) by commercial software COMSOL 6.2 was employed in the wavelength domain. As shown in Figure 4, the normalized electric field distribution varies with θ and ϕ, demonstrating obvious angular sensibility due to the resonance in the structure, confirming its functionality for 3D light-direction sensing.

### 2.3. Deep Learning for Data Fitting

DL offers exceptional capabilities for multidimensional data fitting and inference. According to the universal approximation theorem [27,28,29,30], artificial neural networks used for DL can approximate any function under certain conditions. For the proposed angle sensor, modeling the physical relationship between the (θ,ϕ) and several response variables to calculate the angles from these corresponding data is challenging using traditional methods. In detail, the interdependence between θ and ϕ will lead to an expansion of response data dimensions beyond the computing and fitting ability of traditional analysis methods. DL can address this challenge due to its ability to fit high-dimensional data, and it can even potentially enhance both accuracy and the detection range.

To acquire data for DL model training, the proposed sensors are modeled in simulation software and the response photocurrent quantity on each segment is predefined according to Formula (2) as target data. Within the set detection range of θ and ϕ, parameterized scanning with a step size of 1° is deployed to create a dataset, and the data volume is the product of the detection ranges. Furthermore, the dataset is divided into a training set and a testing set with a ratio of 9:1 in mount to jointly optimize the model.

In this work, a fully connected neural network (FCNN) with three hidden layers and a width of 512 neurons per layer was employed, utilizing the rectified linear unit (ReLU) as the activation function. After the sufficient training of 5000 epochs, the DL model could accurately fit and infer θ and ϕ from the response data generated by the sensor structure. The fitting accuracy during the training was measured by the mean squared error (MSE), while the model’s performance was quantified by the absolute error (ABS) between the calculated 3D light-direction angles and the targets using a pre-divided test dataset.

### 2.4. Fabrication Process

**Figure 5:**
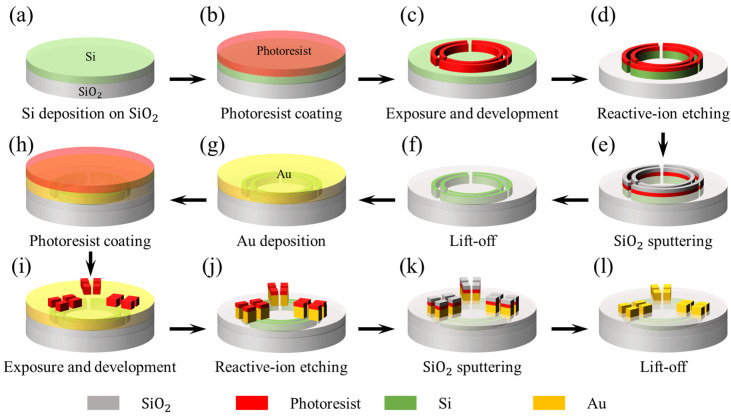
Schematic of the fabrication procedure for the segmented concentric nanorings. (**a**) A 100 nm thick Si on SiO_2_ substrate. (**b**) Photoresist coating. (**c**) Electron beam exposure and development. (**d**) Reactive ion etching. (**e**) SiO_2_ sputtering. (**f**) Lift-off. (**g**) Au deposition. (**h**) Photoresist coating. (**i**) Electron beam exposure and development. (**j**) Reactive ion etching. (**k**) SiO_2_ sputtering. (**l**) Lift-off.

The fabrication of the segmented concentric nanorings included electron beam lithography (EBL), deposition, and lift-off, as shown in Figure 5. Specifically, firstly, a 100 nm thick, lightly n-doped poly-Si layer was deposited on the SiO_2_ substrate using low-pressure chemical vapor deposition (LPCVD). Then, after the photoresist coating, EBL is employed to define the mask patterns followed by dry etching to create the segmented Si nanorings. Subsequently, a 100 nm thick SiO_2_ layer is deposited to cover the nanorings. After lifting off the photoresist, similar processing steps are used to fabricate Au contacts. EBL is used to define the contact patterns, followed by electron-beam physical vapor deposition of Au. The resulting electrodes extend beyond the sensor, facilitating circuit wiring and integration.

## 3. Results

In the simulation, the three-segment nanorings shown in Figure 3 represent the minimum segment configuration required to achieve full azimuthal detection. The width (w) and height (h) of the nanorings are both 100 nm, and the spacing (d) between the inner and outer nanorings within the same pair is 100 nm. The inner radius of the nanoring (r) is 1000 nm, and the central angle (g) corresponding to the gap between adjacent nanoring pairs is 5°.

**Figure 6:**
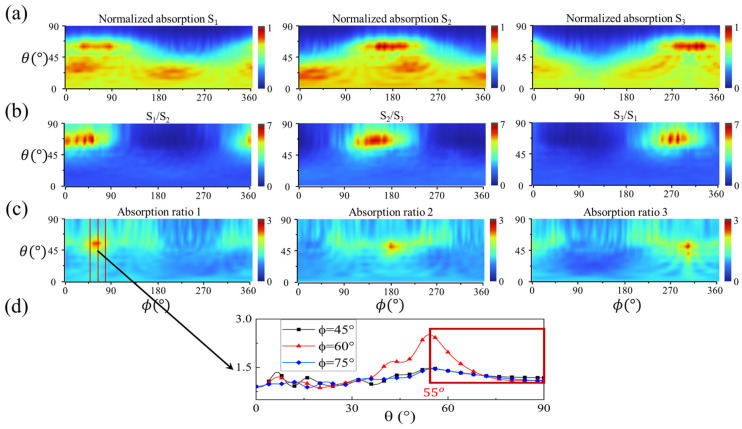
The response of the 3-segment concentric nanoring sensor to the incident light direction angles, θ and ϕ. (**a**) The normalized numerical maps of the total absorption of the *S*_1_, *S*_2_, and *S*_3_ nanoring pairs, including both the inner and outer nanoring segments within each pair. (**b**) The numerical maps of the ratio of the total absorption between adjacent nanoring pairs, *S*_1_/*S*_2_, *S*_2_/*S*_3_, and *S*_3_/*S*_1_. (**c**) The numerical maps of the ratio of the absorption between the inner and outer nanoring segments within each pair, ratio 1, ratio 2, and ratio 3 (e.g., ratio 1 is for the *S*_1_ pair). (**d**) For specific ϕ values, 45°, 60°, and 75°, the absorption ratio for each pair decreases monotonically concerning θ within the range of (55°,90°), which is the computable range for θ.

To elucidate the angular dependence of absorption, detailed absorption maps of the sensor under different incident θ and ϕ at the wavelength (λ) of 550 nm are presented in Figure 6. The results clearly show that the normalized total absorption of the inner and outer nanoring segments within the same pair (Figure 6a), the ratio of total absorption between adjacent nanoring pairs (Figure 6b), and the ratio of absorption between the inner and outer nanoring segments within each pair (Figure 6c) all exhibit clear sensitivity to both θ and ϕ within a specific range of θ (Figure 6d).

From Figure 6d, it can be observed that the detection range for θ, i.e., the monotonic range for a specific ϕ that can be fitted by DL, is approximately 35° (55°∼90°), which was the target range for subsequent calculation. The detection range for θ can be converted and adjusted through a waveguide, where the light in the waveguide obeys the total internal reflection larger than the critical angle. Additionally, due to the translational relationship of responsivities with respect to ϕ between different pairs, the detection range for ϕ is expected to be a full 360°.

Meanwhile, due to the symmetry and translation properties of the proposed structure and its responses as shown, analyzing 1/3 of the sensor is sufficient to verify the overall functionality. We took ϕ within (60°,180°) as an example, whose calculation method is the same as the other ranges, and θ within the computable range of (55°,90°), as illustrated earlier. The FCNN is employed to achieve DL, with the training data inputs consisting of *S*_1_/*S*_2_, *S*_2_/*S*_3_, *S*_3_/*S*_1_, ratio 1, ratio 2, ratio 3, and the targets including θ and ϕ.

Based on the test results, the average ABS for θ is 0.372°, with a maximum of 2.077°, and for ϕ, the average ABS is 0.35°, with a maximum of 2.55°. The sensor demonstrates consistent detection capabilities for both θ and ϕ within its detection range, and its performance in (θ,ϕ) calculation is sufficient for most application scenarios.

## 4. Discussion

### 4.1. Three-Dimensional Light-Direction Angles Calculation

**Figure 7:**
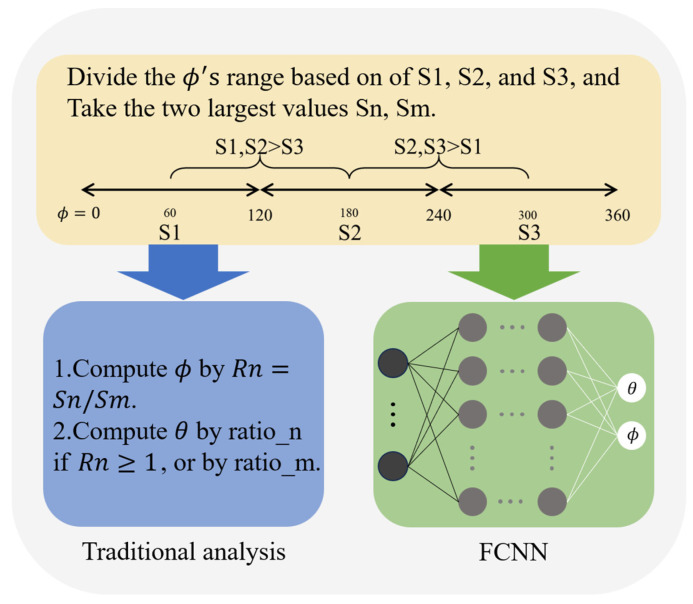
The approach for 3D light-direction angles analysis and calculation. First, an initial division of the azimuth can be made according to the numerical values of the segmented nanoring responses, which helps to select the data sources for accurate (θ,ϕ) calculation. Then, traditional analysis methods or artificial neural networks may be used to compute the θ and ϕ accurately. The former is not feasible because traditional methods are unable to deal with multidimensional data, and we consider using the FCNN to fit the relationship between the responses and the (θ,ϕ), especially the interdependence between the θ and ϕ.

Based on the angle detection principle of the proposed segmented nanorings, θ can be calculated by the RPS and ϕ can be calculated by the RPP. As shown in Figure 7, the precise (θ,ϕ) can be determined based on the corresponding responses, which exhibit high sensitivities to θ and ϕ.

To compute the angles from the responses of the structure, we follow these two steps below: Firstly, we preliminarily divide the range of incident azimuth (ϕ) into 120° steps based on the response amplitudes of the segmented nanoring pairs. This is because the coupling in the pair centered towards the incident light will be the strongest, as shown in Figure 6a. Traditionally, θ might be calculated by RPS and ϕ by RPP independently. However, due to the interdependence between θ and ϕ, this approach is not feasible. To address this issue, we use a FCNN for DL, which treats θ and ϕ as a combined problem, fitting and inferring them from all responses with high precision and a wide-sensing range. This method overcomes the limitations of traditional techniques and leverages the powerful data-fitting capabilities of DL to achieve accurate $\left(\theta ,\phi \right)$ measurements.

### 4.2. More Segments to Improve Detection

**Figure 8:**
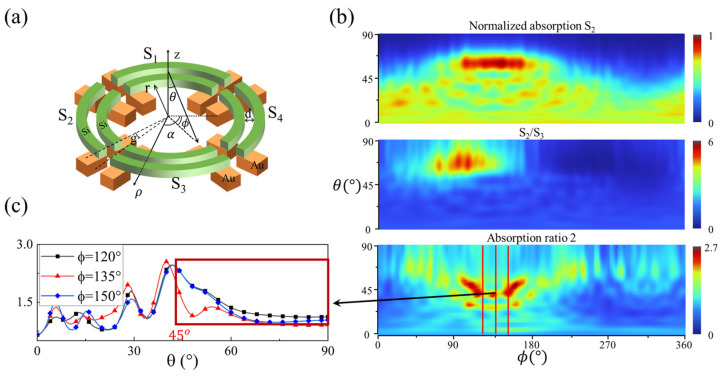
The structural diagram and response of the 4-segment nanorings sensor. (**a**) The structure of the 4 segments. The nanorings are segmented into four pairs—*S*_1_, *S*_2_, *S*_3_, and *S*_4_—with the same structural parameters as the 3-segment structure. In detail, the inner radius of the nanoring (r) is 1000 nm, the width (w) and height (h) of nanorings and the spacing (d) are 100 nm, and the gap between adjacent nanoring pairs corresponds to a central angle (g) of 5°. (**b**) The response of the 4-segment structure. As with the 3 segments, the responses of the 4 segments also show translational symmetry. We present a normalized numerical map of the total absorption of the *S*_2_ pair, the numerical map of the ratio of *S*_2_ and *S*_3_, and the corresponding absorption ratio of the inner and outer rings within *S*_2_ (ratio 2) as examples. (**c**) From the characteristics of ratio 2, it can be seen that the monotonical range of θ has expanded to approximately (45°,90°), which is the computable range for θ.

The number of segments in the proposed sensor significantly impacts the accuracy of the angles’ calculation due to its effect on coupling among the nanoring segments. Increasing the number of segments may enhance both the range and accuracy of the (θ,ϕ) detection. Following the same method, four-segment concentric nanorings with the same structural parameters as the three-segment structure were simulated and tested (Figure 8a), with the results partially presented in Figure 8b as examples. Shown as in Figure 8c, the absorption ratio result shows that the detection range for θ was expanded to approximately 45° (45°∼90°), where the absorption ratio descends monotonically with θ for specific ϕ values.

As with the 3-segment structure, a DL network is used to fit the response data and determine the 3D light-direction angles for the 4-segment configuration. As shown in the results, the average ABS for θ is 0.187°, with a maximum of 2.252°, and for ϕ, the average ABS is 0.174°, with a maximum of 1.704°, indicating that the angular detection capability has been further improved to be better than that of the 3 segments due to more segments being involved. However, it is important to note that although increasing the number of segments may improve the detection range and accuracy, it also increases manufacturing difficulty and costs; thus, a trade-off needs to be considered.

### 4.3. Cross-Arranged Nanowire Pairs for 3D Light-Direction Sensing

**Figure 9:**
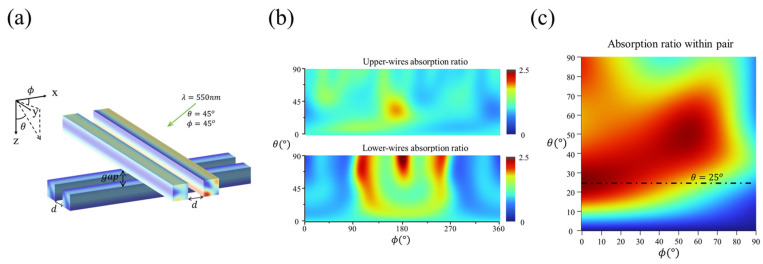
The structure and response of the cross-arranged nanowires sensor to the (θ,ϕ). (**a**) Two pairs of vertically cross-arranged coupled nanowires, with a vertical distance (gap) of 100 nm. The nanowires have a width and height of 100 nm, a length of 500 nm, and an internal spacing (d) of 100 nm within each nanowire pair. (**b**) The numerical maps of the absorption ratio within the upper and lower nanowire pairs with a gap of 100 nm concerning θ and ϕ. The response of the upper layer is different from that of the lower layer, and they do not exhibit a consistent regularity over a wide range of angles when computing the angles. (**c**) The numerical map of the absorption ratio between the two nanowires within a pair when the gap is 1 μm. When the gap is large enough (gap≫λ), the two pairs can be analyzed separately, and their responses have angular symmetry. According to the map, the absorption ratio within a pair is monotonically changing concerning θ for a specific ϕ when θ∈(0,25°), and this range is the detection range for θ.

Additionally, the combination of resonant nanowires can be intuitively expected to expand the detection dimension and function of an angle sensor (Figure 9a). In the simulation, nanowires with a width and height of 100 nm and a length of 500 nm, wrapped in SiO_2_, were divided into two pairs that were internally coupled and arranged in a vertical cross configuration with a specified distance (gap) between them.

With a gap of 100 nm, as shown in Figure 9b, the response of the composite structure for different θ and ϕ does not exhibit a consistent regularity over a wide range, making it impossible to accurately reconstruct large-range 3D light-direction angles. The response of the upper layer differs from that of the lower layer, which is likely due to the interference of the upper-layer nanowires with the incident light, causing the light received by the lower-layer nanowires to differ from the original incident light.

To eliminate the interference between the upper layer and the lower layer, when the gap is large enough (gap≫λ), taking a gap of 1 μm as an example, the response of the two pairs becomes symmetrical. The absorption ratio between the two nanowires within a pair is shown in Figure 9c, exhibiting regular characteristics within a certain range of θ∈(0,25°). In this range, the RPN changes monotonically with respect to θ for a specific ϕ, enabling the calculation of the (θ,ϕ) via DL.

Using the same DL fitting method as before for θ∈(0,25°), and taking ϕ∈(0,45°) as an example that represents the entire structure because of its symmetry, the average ABS for θ is 0.198°, with a maximum of 0.578°, and that for ϕ is 0.948°, with a maximum of 16.387° in the test. It is clear that although the cross-arranged nanowire-pairs can detect (θ,ϕ) when the distance between the upper layer and the lower layer is sufficiently large, the calculation error for ϕ is significantly higher than for θ, which indicates that the sensitivity of the device response to these two angles is inconsistent, rendering it impractical for applications requiring uniform sensitivity.

By contrast, the proposed segmented nanorings, with their segmented and circular characteristics, can sense 3D light-direction angles with a smaller thickness, a wider detection range, and uniform angular sensitivity (Table 1), which demonstrates that the segmented nanoring structure offers better and more stable angle detection capabilities and a wider detection range than the cross-arranged nanowire pairs. The configuration of the segmented concentric nanorings ensures consistent detection capabilities for both θ and ϕ, making it more suitable for practical applications, and it can be inferred that more segments will lead to further improvement.

The FCNNs deployed for these three sensor structures have the same configuration and training parameters, except for the different numbers of input layer neurons due to the varying response amounts of different structures (6, 8, 4, respectively). In detail, the FCNNs consist of three hidden layers with a width of 512 and one output layer with a width of 2 (512 × 512 × 512 × 2), and they are trained for a total of 5000 epochs based on an Adam optimizer, with a learning rate of $10^{-4}$ using the ReLU as the activation function.

**Figure 10:**
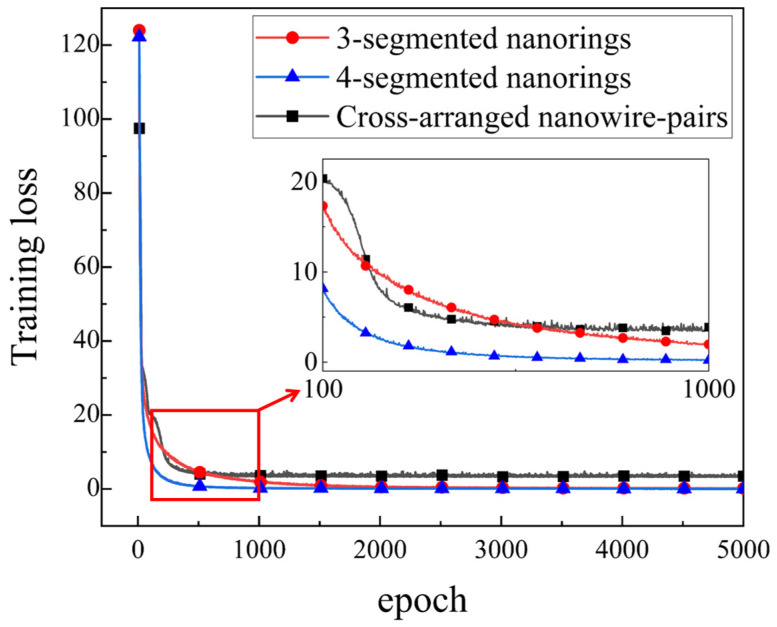
The loss descent curve during the training process of deep learning models corresponding to different structures. (To distinguish clearly, the first 10 epochs corresponding to the loss have been taken out).

As shown in Figure 10, compared to the cross-arranged nanowire pairs structure, the segmented nanorings have smaller convergence errors, and dividing into more segments can result in faster convergence speeds and smaller losses during the DL training process. These improvements may stem from the expansion of response data dimensions brought about by additional segments and the stronger resonance caused by smaller nanoring lengths, which provide computational benefits such as cross-validation between these data in the DL model.

## 5. Conclusions

To achieve precise 3D light-direction angle detection with a single sensing unit, we propose a structure of segmented concentric nanoring pairs absorptive resonators as a possible sensor design approach. In the simulations, this structure exhibits strong and uniform angular sensitivity to both elevation and azimuth angles. By employing deep learning for data fitting, the sensor can achieve a detection range of 0∼360° for azimuth and 45°∼90° for elevation, with an average accuracy of 0.19° in (θ,ϕ) angle sensing, thereby meeting the requirements of most application scenarios. The accuracy and range of angle sensing can be improved with an increase in the number of segments. Finally, based on the structure of segmented concentric nanorings, by combining the resonance characters with deep learning, precise angle sensing is possible, showing various promising applications such as in optical field reconstruction, dynamic ranging, and optical interaction, and even military reconnaissance and guidance could be achieved by array integration. It should be pointed out that the proposed sensor was only validated through simulations, but we believe that through further exploration and experimentation, practical applications will be inspired to come to fruition in the near future.

## Figures and Tables

**Table 1:** The absolute error of θ and ϕ for different angular detection structures, including the detection range for θ (the detection ranges for ϕ are all 360°), average absolute error, maximum absolute error, and the variance.

Structure	Detection Range for θ (°)	Absolute Error of θ (°)			Absolute Error of ϕ (°)		
		Average	Maximum	Variance	Average	Maximum	Variance
3-segment nanorings	55–90	0.372	2.077	0.121	0.350	2.550	0.102
4-segment nanorings	45–90	0.187	2.252	0.041	0.174	1.704	0.030
Cross-arranged nanowire pairs	0–25	0.198	0.578	0.009	0.948	16.387	7.291

## Data Availability

The raw data supporting the conclusions of this article will be made available by the authors on request.
